# The Effects of Petroleum Emanations on Health

**Published:** 1887-01

**Authors:** 


### *THE EFFECTS OF PETROLEUM EMANATIONS ON HEALTH.*

Wielazyk has made observations upon the workmen in the Carpathian oil regions. Crude petroleum is a thick, oily, greenish-brown liquid, composed of a mixture of gaseous hydro-carbons, liquids and solids. The workmen about the wells are exposed to an atmosphere corrupted by the presence of marsh gas, carbonic acid, ithylem, hydro-carbons, carbon non-oxide, and often sulphurated hydrogen. Cases of asphyxia are not rare. The effects of long exposure to air thus contaminated are tinnitus, luminous circles before the eyes, throbbing of the temples, loss of consciousness and hallucinations. These last are frequent. One workman will hear a voice ordering him to remain at the bottom of the well, another picks up stones believing them to be gold. The action of the gases resembles somewhat the opiates. A workman slept six hours at the bottom of a well, and was angry on being awakened from a sleep so agreeable. Diseases are rare among the workmen, acne is sometimes observed, but affections of the respiratory organs are almost unknown. Phthisis was found in only two of six hundred workmen, although many of them had a hereditary tendency to the disease. The author explains the infrequency of phthisis to the disinfectant action of petroleum emanations. Skin diseases are also rare.—*Bull. Gen. de Therapeutique.*

